# Supplementary material for: Aggregation of Italian Lichen Data in ITALIC 7.0
Source: J Fungi (Basel). 2023 May 11;9(5):556. doi: 10.3390/jof9050556 (PMC10219532; doi:10.3390/jof9050556)
Supplement: Supplementary file 1 [file jof-09-00556-s001.zip › supplementary1.html]

Javascript must be enabled to view this page.

magnitude

Erbari

 87826

 87826

 80178

 70548

 3943

 146

 9

 2

 9

 4

 70

 52

 373

 1

 9

 8

 9

 2

 13

 11

 4

 255

 20

 23

 4

 2

 1

 3

 8

 905

 1

 1

 11

 1

 420

 2

 1

 4

 3

 6

 282

 1

 1

 5

 3

 6

 28

 1

 1

 73

 1

 53

 5

 5

 81

 3

 78

 285

 285

 585

 547

 14

 24

 345

 345

 1131

 1128

 3

 33

 33

 21

 3

 18

 33

 33

 1098

 1098

 720

 4

 11

 3

 27

 116

 214

 3

 8419

 2887

 134

 1

 1

 177

 580

 1146

 84

 220

 310

 24

 3

 38

 30

 31

 2

 81

 22

 3

 5532

 425

 16

 134

 229

 48

 12

 684

 38

 1817

 96

 1163

 2

 803

 26

 39

 34201

 4885

 26

 3

 431

 253

 8

 9

 135

 10

 190

 28

 48

 21

 2

 18

 37

 397

 60

 74

 12

 1

 2192

 29

 428

 1

 113

 304

 55

 12175

 1

 152

 4

 5

 5

 28

 8

 103

 10

 378

 4

 10

 1129

 1

 92

 1

 7

 68

 11

 15

 572

 2

 637

 59

 741

 124

 52

 7

 130

 76

 6

 72

 739

 392

 59

 41

 9

 362

 2

 8

 3

 1

 6

 8

 1132

 710

 2

 266

 710

 237

 242

 184

 4

 116

 530

 1

 309

 1

 5

 1

 759

 826

 130

 1

 14

 6

 5

 104

 1863

 4

 3

 3

 2

 811

 30

 5

 623

 56

 5

 1

 299

 3

 1

 6

 11

 10

 10

 609

 15

 4

 277

 19

 289

 5

 5540

 4

 16

 4

 1

 19

 3130

 1011

 78

 679

 1

 352

 22

 73

 138

 12

 1010

 46

 50

 910

 4

 17

 17

 574

 369

 34

 171

 5508

 2

 17

 5425

 1

 7

 51

 5

 1

 1

 87

 87

 1527

 2

 685

 415

 424

 1

 2

 2

 3

 2

 1

 44

 44

 24

 24

 192

 192

 155

 155

 8

 2

 142

 3

 6254

 6234

 1

 401

 2

 422

 71

 231

 682

 2

 11

 4

 355

 6

 552

 24

 239

 155

 12

 6

 71

 192

 579

 77

 258

 64

 92

 10

 25

 7

 126

 3

 15

 339

 7

 221

 178

 794

 6

 6

 2

 2

 3

 3

 9

 9

 1536

 1517

 3

 47

 94

 7

 155

 80

 25

 11

 553

 61

 1

 55

 1

 2

 311

 90

 1

 18

 2

 19

 19

 596

 469

 1

 1

 19

 113

 5

 120

 210

 127

 44

 1

 1

 81

 223

 125

 3

 10

 1

 65

 46

 85

 85

 13

 13

 3237

 1026

 168

 467

 246

 132

 13

 9

 7

 2

 346

 99

 3

 91

 22

 131

 1093

 3

 1090

 2

 2

 761

 611

 150

 6763

 2044

 44

 29

 496

 296

 323

 293

 3

 9

 27

 524

 20

 12

 8

 1153

 31

 5

 442

 2

 76

 2

 169

 255

 166

 2

 3

 939

 2

 2

 192

 4

 1

 6

 114

 144

 285

 73

 93

 1

 22

 98

 93

 2

 3

 64

 36

 9

 19

 421

 421

 1798

 1493

 305

 200

 200

 26

 26

 1600

 203

 1

 152

 50

 5

 5

 1259

 1

 2

 1238

 18

 129

 97

 4

 28

 4

 4

 1248

 1217

 295

 912

 10

 31

 31

 945

 875

 10

 5

 2

 858

 70

 68

 2

 26

 26

 23

 2

 1

 89

 89

 3

 86

 153

 153

 80

 27

 46

 62

 34

 34

 28

 28

 1186

 350

 350

 284

 66

 127

 127

 37

 1

 55

 1

 2

 1

 19

 2

 1

 7

 1

 15

 15

 15

 146

 36

 36

 4

 4

 106

 106

 60

 55

 55

 5

 5

 397

 397

 53

 4

 286

 54

 91

 91

 57

 34

 3070

 2752

 2746

 129

 8

 225

 112

 3

 289

 99

 2

 3

 73

 33

 1

 3

 1

 2

 267

 31

 8

 38

 136

 40

 74

 142

 7

 17

 167

 175

 625

 36

 6

 6

 262

 262

 1

 3

 20

 236

 2

 3

 3

 3

 3

 3

 3

 50

 50

 50

 3428

 3428

 355

 244

 71

 21

 19

 1242

 887

 6

 142

 6

 6

 2

 2

 142

 45

 3

 1

 125

 76

 43

 6

 1175

 3

 168

 2

 166

 21

 251

 1

 21

 25

 18

 14

 89

 5

 281

 3

 2

 80

 8

 17

 173

 173

 351

 9

 5

 11

 2

 316

 1

 4

 3

 7

 1

 6

 542

 542

 431

 80

 1

 4

 18

 1

 3

 38

 1

 1

 20

 5

 68

 17

 28

 4

 2

 25

 17

 10

 13

 45

 1

 3

 21

 5

 6

 6

 105

 105

 126

 86

 6

 4

 2

 48

 16

 1

 15

 16

 32

 32

 35

 35

 3

 32

 5

 5

 5

 1267

 1267

 1267

 1182

 85

 10

 10

 10

 3

 7

 75

 75

 14

 14

 14

 54

 54

 10

 12

 32

 7

 7

 7

 7573
